# Attenuated *Salmonella* carrying siRNA-PD-L1 and radiation combinatorial therapy induces tumor regression on HCC through T cell-mediated immuno-enhancement

**DOI:** 10.1038/s41420-023-01603-x

**Published:** 2023-08-28

**Authors:** Huijie Jia, Pengkun Wei, Shijie Zhou, Yuanyuan Hu, Chunjing Zhang, Lirui Liang, Bingqing Li, Zerui Gan, Yuanling Xia, Hanyu Jiang, Mingguang Shao, Sheng Guo, Zishan Yang, Jiateng Zhong, Feng Ren, Huiyong Zhang, Yongxi Zhang, Tiesuo Zhao

**Affiliations:** 1 Department of Oncology, The Third Affiliated Hospital of Xinxiang Medical University, 453000 Xinxiang, Henan P. R. China; 2 Xinxiang Engineering Technology Research Center of Immune Checkpoint Drug for Liver-Intestinal Tumors, Xinxiang Medical University, 453000 Xinxiang, Henan P. R. China; 3 Department of Pathology, School of Basic Medical Sciences, Xinxiang Medical University, 453000 Xinxiang, Henan P. R. China; 4 Department of Immunology, School of Basic Medical Sciences, Xinxiang Medical University, 453000 Xinxiang, Henan P. R. China; 5 Henan International Joint Laboratory of Immunity and Targeted Therapy for Liver-Intestinal Tumors, Xinxiang Medical University, 453000 Xinxiang, Henan P. R. China; 6 Synthetic Biology Engineering Lab of Henan Province, School of Life Science And Technology, Xinxiang Medical University, 453000 Xinxiang, Henan P. R. China

**Keywords:** Tumour immunology, Immunotherapy

## Abstract

Hepatocellular carcinoma (HCC), the most prevalent type of aggressive liver cancer, accounts for the majority of liver cancer diagnoses and fatalities. Despite recent advancements in HCC treatment, it remains one of the deadliest cancers. Radiation therapy (RT) is among the locoregional therapy modalities employed to treat unresectable or medically inoperable HCC. However, radioresistance poses a significant challenge. It has been demonstrated that RT induced the upregulation of programmed death ligand 1 (PD-L1) on tumor cells, which may affect response to PD-1-based immunotherapy, providing a rationale for combining PD-1/PD-L1 inhibitors with radiation. Here, we utilized attenuated *Salmonella* as a carrier to explore whether attenuated *Salmonella* carrying siRNA-PD-L1 could effectively enhance the antitumor effect of radiotherapy on HCC-bearing mice. Our results showed that a combination of siRNA-PD-L1 and radiotherapy had a synergistic antitumor effect by inhibiting the expression of PD-L1 induced by radiation therapy. Mechanistic insights indicated that the combination treatment significantly suppressed tumor cell proliferation, promoted cell apoptosis, and stimulated immune cell infiltration and activation in tumor tissues. Additionally, the combination treatment increased the ratios of CD4^+^ T, CD8^+^ T, and NK cells from the spleen in tumor-bearing mice. This study presents a novel therapeutic strategy for HCC treatment, especially for patients with RT resistance.

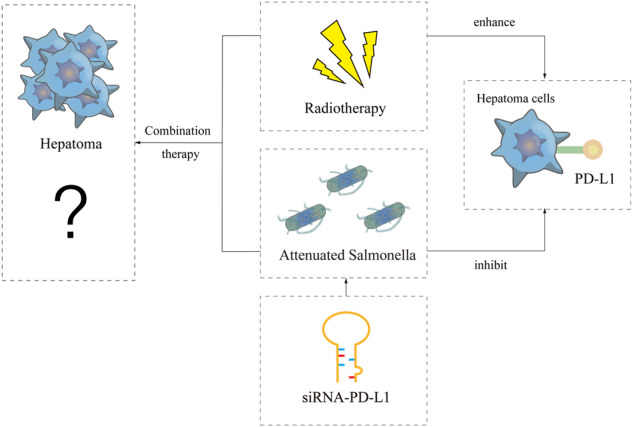

## Introduction

As a prevalent malignant tumor affecting the digestive tract, liver cancer ranks as the third most common cause of cancer-related deaths worldwide [1]. Hepatocellular carcinoma (HCC) accounts for 75 to 85 percent of primary liver cancer diagnoses. Over 500,000 new cases of HCC are reported annually, with 50% occurring in China [2]. Although surgical resection, radiofrequency ablation, and liver transplantation are potentially curative treatments for early-stage HCC [3], recurrence rates within 5 years remain over 60% for patients who have undergone tumor resection or radiofrequency ablation [4, 5]. Therefore, the management of HCC remains a pressing global health challenge.

The increasing application of radiation therapy (RT) has highlighted its crucial role in HCC treatment [6]. However, despite its effectiveness, some patients still experience local recurrence and distant metastasis following radiotherapy [7]. The upregulation of PD-L1 expression after radiotherapy has been found to negatively regulate the antitumor immune response of T cells by interacting with the PD-1 protein, leading to the expression of an immune-negative regulatory axis that promotes HCC invasion [8–10]. This may explain the limited efficacy of radiotherapy. PD-L1, an immune-negative molecule, is highly expressed on the surface of tumor cells [11, 12] and binds with PD-1 on T cells, resulting in the inhibition of T cell-mediated killing of tumor cells [13] and promoting tumor progression [14]. Therefore, blocking the PD-1/PD-L1 pathway has emerged as a vital strategy for HCC treatment.

The signaling pathway can be obstructed through two methods: either at the protein level using monoclonal antibody [15] or at the gene level using RNA interference (RNAi) technology. Patisiran, the world’s first RNAi drug, was approved by the U.S. Food and Drug Administration in 2018 and essentially cured hereditary transthyroxin amyloidosis, or h-ATTR, by silencing the disease-causing gene [16]. Furthermore, RNAi can block the expression of oncogenes within the body while having little effect on the expression of other genes [17]. Several studies have demonstrated that siRNA-PD-L1 can be transported to malignant tumor tissue and impede the interaction of PD-1/PD-L1, thereby exhibiting certain antitumor effects [18–20]. Nevertheless, the administration of siRNA itself encounters challenges such as enzyme degradation, poor cell uptake, and low in vivo efficiency [21]. Therefore, stable delivery carriers are required.

Our prior research uncovered that attenuated *Salmonella* proficiently conveyed siRNA to neoplastic tissue, manifesting an anti-tumorigenic effect [22]. As a transporter, attenuated *Salmonella* has the quality of aggregating in neoplastic tissue. Furthermore, as a facultative anaerobe, *Salmonella* can proliferate not only in large, hypoxic solid tumors but also survive in small metastatic tumors with oxygen [23, 24]. It is worth noting that attenuated *Salmonella* vectors can overcome the diffusion barrier in neoplastic tissue and accomplish targeted tumor treatment by transporting specific genes [25].

In light of the above, our present study aimed to investigate whether attenuated *Salmonella* carrying siRNA-PD-L1 could significantly enhance the antitumor effect of radiotherapy on hepatocellular carcinoma-bearing mice by inhibiting the expression of PD-L1. Our findings may offer novel evidence for ameliorating the clinical resistance of hepatocellular carcinoma patients to radiotherapy.

## Results

### The upregulation of PD-L1 was reversed by the treatment of siRNA-PD-L1 carried by attenuated *Salmonella*

**Fig. 1:**
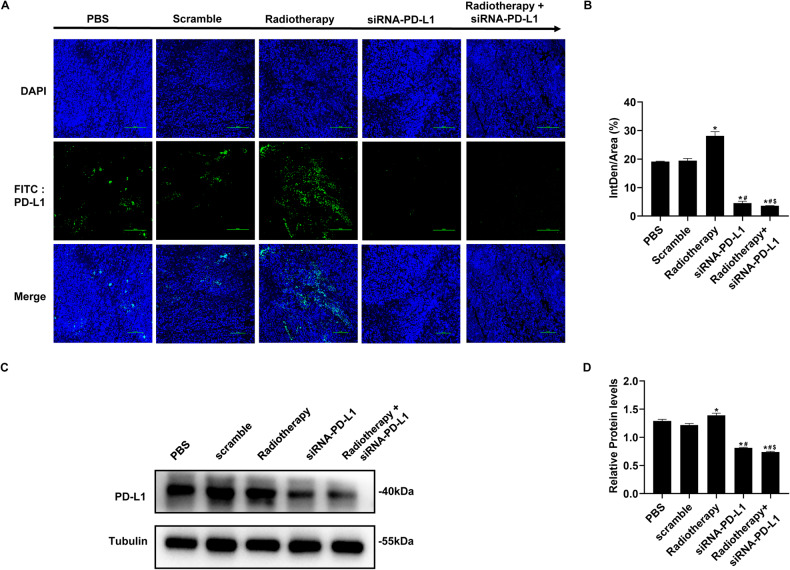
Effects of combination therapy on PD-L1 expression. **A** PD-L1 expression in different treatment groups determined by immunofluorescence (IF). **B** Statistical analysis of the results shown in (**A**). **C** PD-L1 levels in each group assessed by Western blotting (WB). **D** Statistical analysis of the WB results shown in (**C**). The data are expressed as mean ± SD (*n* = 3). *P < 0.05 versus PBS group; #*P* < 0.05 versus Scramble group; $*P* < 0.05 versus Radiotherapy group.

Previous studies have indicated that radiotherapy can increase the expression of PD-L1 on tumor cells, thereby reducing the efficacy of radiotherapy. In malignant diseases, the expression of PD-L1 is closely associated with tumor progression and poor prognosis and is indicative of drug resistance to conventional therapies such as chemotherapy and radiotherapy. Therefore, in order to investigate the effect of the combination therapy on PD-L1 expression, we performed immunofluorescence and Western blot analyses. As demonstrated by Fig. 1, PD-L1 expression was upregulated in tumor tissue after radiotherapy when compared to the PBS group. However, with the remarkable use of attenuated *Salmonella* carrying siRNA-PD-L1, the upregulation of PD-L1 was significantly reversed, suggesting that siRNA-PD-L1 improved the immunosuppressive microenvironment induced by radiotherapy and that the combined treatment was synergistic.

### Synergistic effect of radiotherapy and attenuated *Salmonella*-mediated delivery of siRNA-PD-L1 on tumor growth in xenograft tumor-bearing mice

**Fig. 2:**
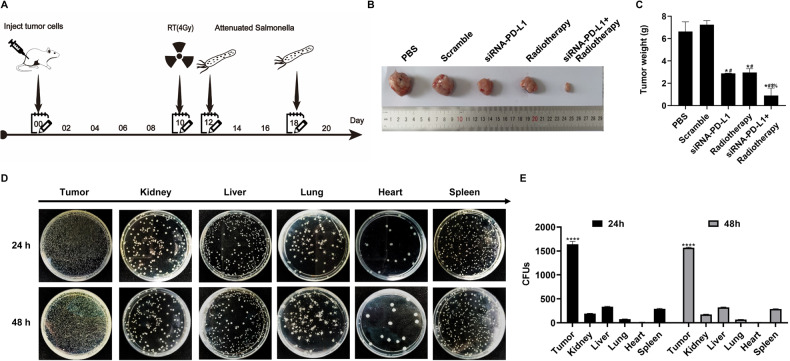
Effects of various treatments on tumor growth. To generate tumors, 2 × 10^6^ H22 cells were subcutaneously inoculated into the flank of C57BL/6 mice. When the tumors reached a mean volume of approximately 100 mm^3^, the mice were divided into five groups and treated differently. **A** Treatment timeline. **B** Representative tumor images of each group. **C** Statistical analysis of tumor weight for each treatment method. **D** Accumulation of attenuated *Salmonella* in different organs. **E** Statistical analysis of (**D**). The data are presented as mean ± SD (*n* = 3). *P < 0.05 versus PBS group; #*P* < 0.05 versus Scramble group; $*P* < 0.05 versus Radiotherapy group; %*P* < 0.05 versus siRNA-PD-L1 group. ****P < 0.0001 compared to the Kidney, Liver, Lung, Heart, and Spleen groups.

Following the establishment of subcutaneous xenograft tumors via the injection of H22 cells, treatment was initiated on day 10 when the tumors reached 100 mm^3^. The mice were subjected to a 4 Gy dose of radiation on day 10 and/or administered attenuated *Salmonella* carrying siRNA-PD-L1 at colony-forming units (CFU) values of 4 × 10^5^ per mouse on day 12 and day 18, respectively, via intraperitoneal injection. Mice were humanely sacrificed on day 7 after the last treatment, and the tumors were meticulously removed, photographed, and weighed (Fig. 2A). The results indicated that compared to the control groups (PBS group and Scramble group), the two monotherapy groups (Radiation therapy group and siRNA-PD-L1 group) significantly inhibited tumor growth. However, the combination group exhibited a superior antitumor effect, as evidenced by a reduction in tumor size and weight (Fig. 2B, C). Furthermore, to determine whether attenuated *Salmonella* was capable of selectively localizing in tumor tissue, we assessed the distribution of Salmonella in the tumor and other organs (kidney, liver, lung, heart, and spleen). As depicted in Fig. 2D, E, the number of *Salmonella* clones was found to be predominantly higher (5- to 120-fold) in tumor tissues compared to other tissues such as kidney, liver, lung, heart, and spleen tissues, implying that attenuated *Salmonella* chiefly accumulates in tumor tissue with minimal toxicity on normal organs.

### Radiotherapy in combination with attenuated *Salmonella* carrying siRNA-PD-L1 significantly suppressed the proliferation of tumor cells in the tumor-bearing mice

**Fig. 3:**
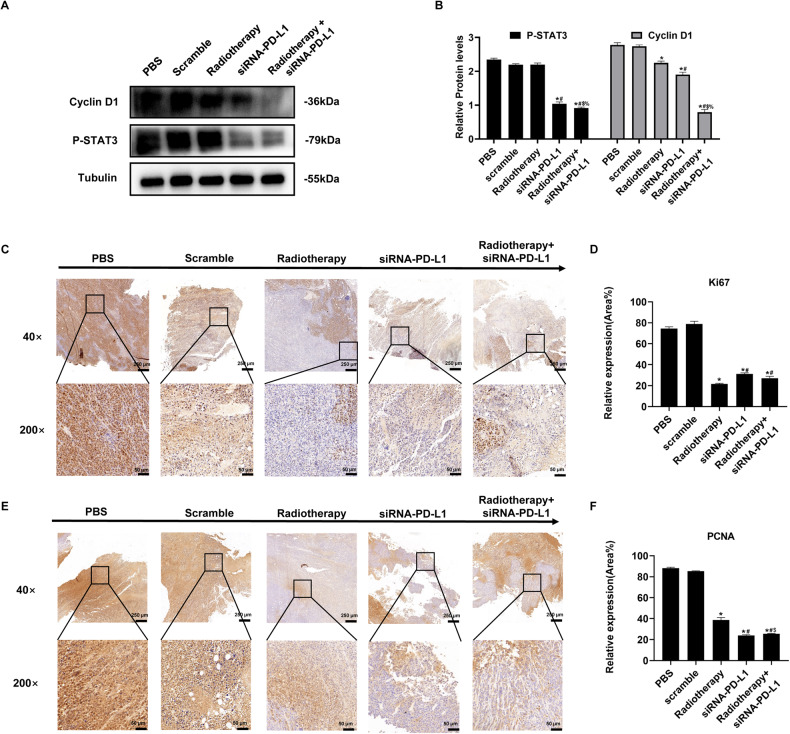
Effects of various treatments on tumor cell proliferation. **A** Western blot analysis of the protein expression levels of proliferation-related proteins. **B** Quantitative analysis of tumor cell proliferation based on the results of (**A**). **C** Immunohistochemistry (IHC) analysis of Ki67+ cells in tumor tissues. **D** Statistical analysis of the IHC results in (**C**). **E** Tumor cell proliferation assessed by PCNA immunohistochemistry. **F** Statistical analysis of the results in (**E**). The data are expressed as mean ± SD (*n* = 3). *P < 0.05 versus PBS group; #*P* < 0.05 versus Scramble group; $*P* < 0.05 versus Radiotherapy group; %*P* < 0.05 versus siRNA-PD-L1 group.

To investigate the specific mechanism underlying the inhibition of tumor growth in tumor-bearing mice by combining radiation therapy with attenuated *Salmonella* carrying siRNA-PD-L1, we analyzed the expression of proliferation-related proteins in tumor tissues. Proliferation-related proteins P-STAT3 and Cyclin D1 in tumor tissues were examined by Western blot analysis. The results indicated that the level of both P-STAT3 and Cyclin D1 was significantly inhibited in the combination group as compared to other groups (Fig. 3A, B). Moreover, this finding was further confirmed by immunohistochemistry (IHC) against the proliferating cell markers Ki67 and proliferating cell nuclear antigen (PCNA) (Fig. 3C–F). These results collectively suggest that the treatment approach of combining radiotherapy with siRNA-PD-L1 can effectively suppress the proliferation of tumor cells in tumor-bearing mice.

### Combined treatment of radiotherapy and siRNA-PD-L1 effectively induced cell apoptosis in tumor-bearing mice

**Fig. 4:**
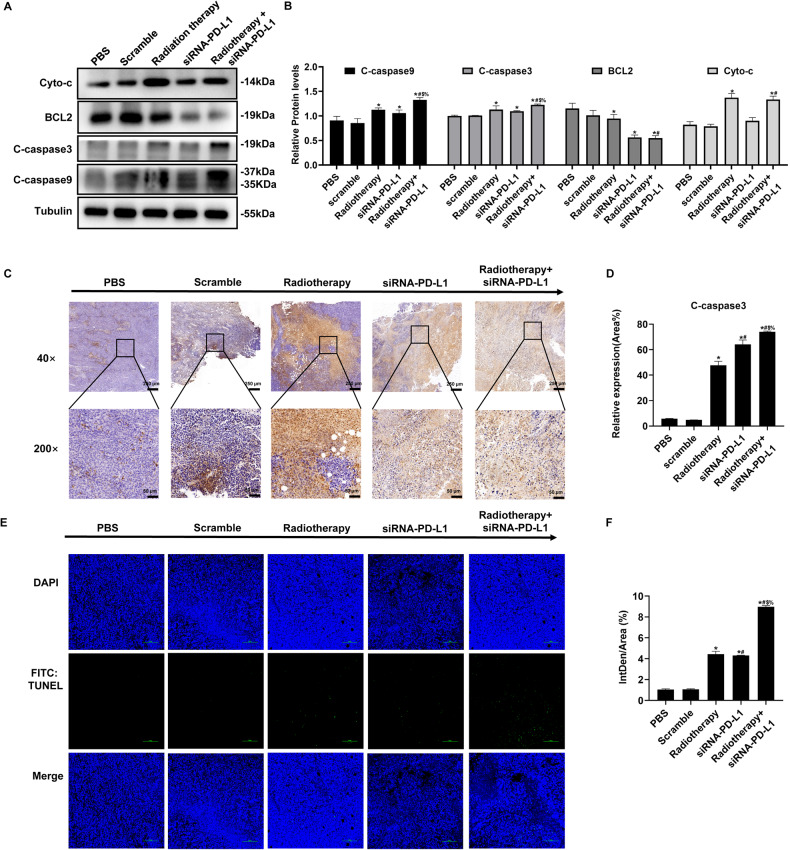
Different treatments on tumor cell apoptosis. Evaluation of apoptotic cells was performed on tumor tissues obtained 16 days after initiation of treatment. **A** Western blot analysis was used to determine the expression levels of apoptosis-related proteins. **B** Statistical analysis of the WB results shown in (**A**). **C** Immunohistochemical analysis of the expression levels of C-caspase3 proteins. **D** Statistical analysis of the IHC graphs in (**C**). **E** Tumor cell apoptosis was detected by TUNEL assay. **F** Statistical diagram of (**E**). The data are expressed as mean ± SD (*n* = 3). *P < 0.05 versus PBS group; #*P* < 0.05 versus Scramble group; $*P* < 0.05 versus Radiotherapy group; %*P* < 0.05 versus siRNA-PD-L1 group.

To investigate the underlying mechanism, we analyzed the expression of apoptosis-related proteins in tumor tissue using Western blot. Caspases are the key enzymes involved in apoptosis, particularly caspase3 and caspase9, which is the main executive enzyme. As shown in Fig. 4A, B, the cleaved caspase9 (C-caspase9) was significantly upregulated in the combination group compared to other groups. This finding was confirmed by the increase in mitochondrial protein Cyto-C and decrease in anti-apoptotic protein Bcl-2 (Fig. 4A, B). Furthermore, we detected the activation of caspase3 by IHC, which showed the same results as C-caspase3 (Fig. 4C, D). The TUNEL assay also revealed a higher apoptotic rate in the combined treatment group (Fig. 4E, F). Therefore, our results indicate that the combined therapy of radiotherapy and siRNA-PD-L1 carried by attenuated *Salmonella* promotes tumor cell apoptosis by inhibiting Bcl-2, which facilitates cyto-c release.

### The co-treatment of radiotherapy and siRNA-PD-L1 resulted in a remarkable increase in the proportion of tumor-infiltrating immune cells in tumor tissues of tumor-bearing mice

**Fig. 5:**
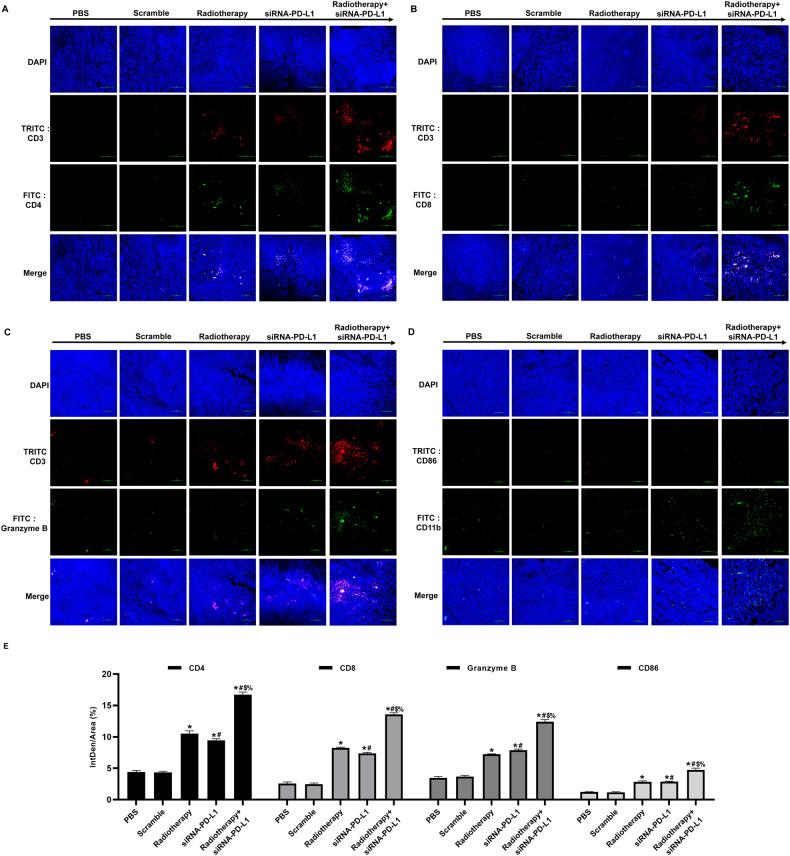
Effects of combination therapy on immune cell infiltration in tumor tissue. **A**–**D** Infiltration of immune cells in tumor tissue detected by immunofluorescence assay. **E** Semi-quantitative statistical analysis of (**A**–**D**). The data are presented as mean ± SD (*n* = 3). *P < 0.05 versus PBS group; #*P* < 0.05 versus Scramble group; $*P* < 0.05 versus Radiotherapy group; %*P* < 0.05 versus siRNA-PD-L1 group.

Programmed cell death 1 ligand 1 (PD-L1) is a co-inhibitory molecule expressed in various cell types, including tumor cells. By engaging PD-1 on T cells, tumor-expressed PD-L1 suppresses the antitumor response by inhibiting T cell activation. Therefore, we assessed the effect of combination treatment on T-helper lymphocytes (CD4^+^), cytotoxic lymphocytes (CD8^+^), activated lymphocytes (Granzyme B^+^), and antitumor macrophages (CD86 positive M1 type macrophages) infiltrating tumor tissue (Fig. 5). The results demonstrated that radiotherapy in combination with attenuated *Salmonella* carrying siRNA-PD-L1 significantly enhanced the infiltration and activation of immune cells in tumor tissue.

### Combination therapy effectively regulated the proportion of immune cells within the spleen

**Fig. 6:**
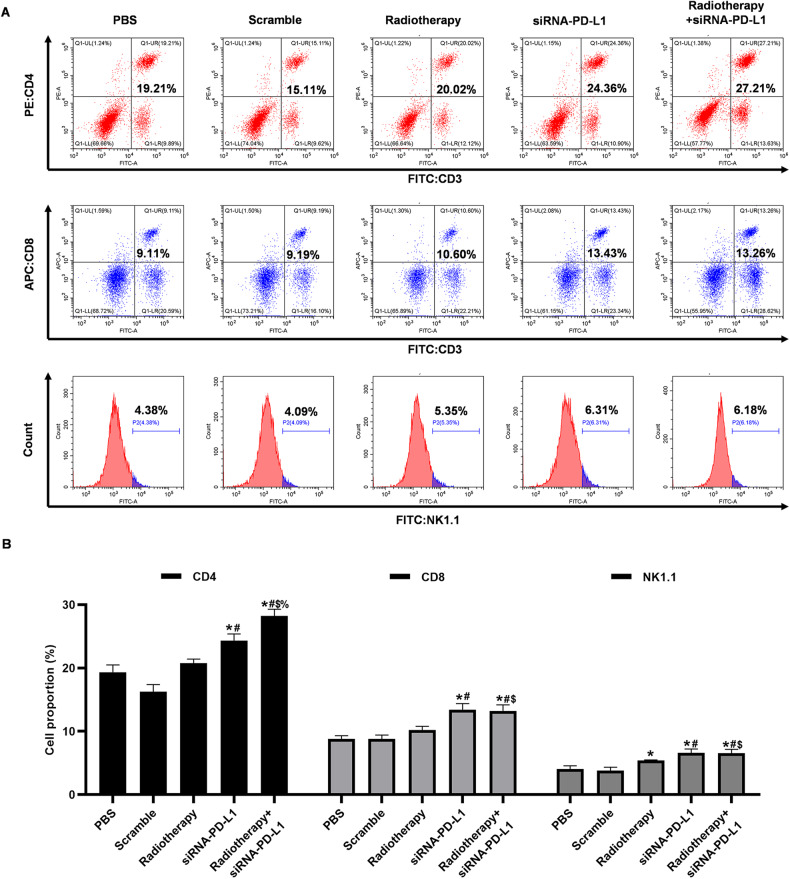
Effects of combination therapy on immune cell distribution in the spleen. **A** Distribution of CD3^+^CD4^+^ T cells, CD3^+^CD8^+^ T cells, and NK1.1+ cells in the spleen from tumor-bearing mice assessed by flow cytometry. **B** Statistical analysis of the results shown in (**A**). The data are presented as mean ± SD (*n* = 3). *P < 0.05 versus PBS group; #*P* < 0.05 versus Scramble group; $*P* < 0.05 versus Radiotherapy group; %*P* < 0.05 versus siRNA-PD-L1 group.

The spleen is the largest peripheral immune organ and a crucial center for immune response in the body. Given its pivotal role in antitumor immunity, we conducted a comprehensive analysis of cell immunity modulation in response to combination treatment via flow cytometry. Our findings indicated a notable increase in the proportion of CD4^+^ T lymphocytes, CD8^+^ T lymphocytes, and NK cells in both single treatment groups (i.e., radiotherapy and siRNA-PD-L1 groups). Significantly, the upregulation of immune cells was considerably more pronounced in the combined treatment group, as revealed by Fig. 6. These results underscore the ability of combination therapy to effectively alter splenic immune status and bolster systemic cellular immunity against tumors.

## Discussion

Radiation therapy has been established as a viable clinical approach for treating HCC [26]. Our study also found that radiation therapy significantly inhibited tumor growth, possibly by inhibiting cell proliferation and increasing apoptosis in tumor cells. However, emerging evidence suggests that radiation therapy may enhance immune escape by inducing upregulation of PD-L1 in hepatocellular carcinoma [8]. Therefore, we demonstrated that combining attenuated *Salmonella* carrying siRNA-PD-L1 with radiation therapy resulted in a significant improvement in the antitumor effect in mice with HCC.

PD-L1, which is expressed in inflamed epithelial cells, tumor cells, stromal cells, and tumor cells, is a promising therapeutic target for cancer treatment. Blocking the PD-L1 pathway can reverse T lymphocyte exhaustion and promote an antitumor response [27–29]. Our data confirmed that PD-L1 expression was higher in the tumor tissue of HCC-burdening mice treated with radiation therapy, which may contribute to radioresistance. In this study, siRNA-PD-L1 carried by attenuated *Salmonella* significantly enhanced the radiosensitivity of HCC by inhibiting PD-L1 expression in tumor tissues. Radiation therapy induces DNA damage in tumor cells, which promotes the release of dsDNA and activates the cGAS/STING pathway, leading to lymphocyte infiltration in tumor tissues [9]. The extent of tumor-infiltrating lymphocytes (TIL) is closely associated with the prognosis of cancer patients [30–32]. Our results also revealed that radiation therapy markedly increased the infiltration of CD8^+^ T lymphocytes in tumor tissues but not the ratios of T lymphocytes in spleens. Surprisingly, radiation therapy increased the level of PD-L1 expression but also increased the number of Granzyme B^+^ T lymphocytes in tumor tissues, indicating that factors other than PD-L1 also inhibit T lymphocyte function. siRNA-PD-L1 carried by attenuated *Salmonella* showed a similar tendency. Importantly, combined treatment not only further increased the ratio of TIL but also the activation of T lymphocytes, confirming that the combination therapy had a synergistic effect on promoting T cell function.

Furthermore, macrophages are important cells involved in innate immunity, and they have been classified into two types: M1 and M2. M1 macrophages exhibit potent antitumor effects, while M2 macrophages contribute to tumor progression, as confirmed by previous studies [33–35]. It has been reported that 2-deoxy-d-glucose significantly enhances the antitumor effect of radiation therapy by promoting the polarization of macrophages toward the M1 type [36]. This suggests that regulating the polarization of macrophages might be an effective strategy to enhance the efficacy of radiation therapy. Klug et al. also discovered that low-dose radiation can enhance cell differentiation toward M1 macrophages and optimize T lymphocyte function [37]. In this study, radiation therapy with a dose of 4 Gy increased the infiltration of M1 macrophages in tumor tissues, which might have contributed to the activation of T lymphocytes. However, PD-L1 upregulation dramatically impaired the polarization of M1 macrophages [38]. Therefore, treatment with siRNA-PD-L1 significantly enhanced the infiltration of M1 macrophages by downregulating PD-L1 expression. Moreover, the combination of siRNA-PD-L1 and radiation therapy displayed a synergistic effect on the polarization of M1 macrophages.

**Fig. 7:**
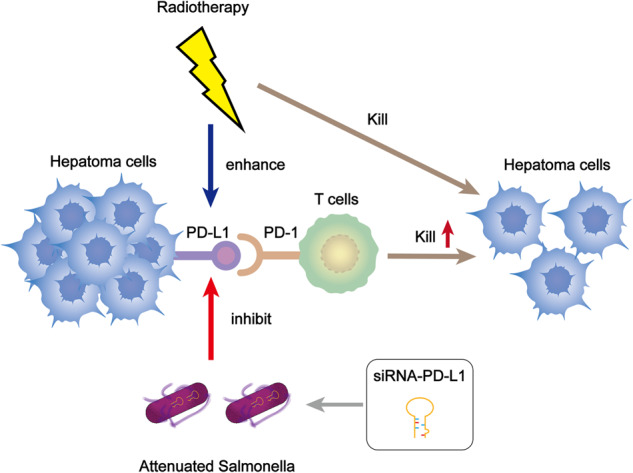
Therapeutic effectiveness of combination treatment in tumor-bearing mice. The effectiveness of radiation therapy is reduced due to the overexpression of PD-L1 on the surface of tumor cells post-treatment. However, by utilizing attenuated *Salmonella* that carries siRNA-PD-L1 and combining it with radiation therapy, the function of T lymphocytes is restored, resulting in enhanced efficacy of radiation therapy and a synergistic antitumor effect.

In summary, our study demonstrated that the combination of siRNA-PD-L1 and radiation therapy exhibited a synergistic antitumor effect by inhibiting the upregulation of PD-L1 induced by radiation therapy (Fig. 7). This finding suggests a promising combination strategy for the treatment of HCC, particularly for patients with radioresistance.

## Materials and methods

### Cell lines and strains

The Mouse H22 hepatoma cells are stored in the Xinxiang Key Laboratory of Tumor Vaccine and Immunotherapy at Xinxiang Medical CollegePage, located in Henan Xinxiang 453000, P.R. China. The cell lines were tested for mycoplasma contamination by PCR-based method and confirmed negative for mycoplasma. The attenuated *Salmonella* typhimurium and strains carrying Scramble and siRNA-PD-L1 plasmids were previously constructed and are also stored in the same laboratory [39].

### Establishment of tumor-bearing mice model and treatment

Female C57BL/6 mice of SPF grade, aged 6–8 weeks, were procured from Henan Skbex Biotechnology Co., Ltd. and raised under pathogen-free conditions at a temperature of 24 ± 2 °C with a regulated light schedule of 12-h light/dark cycle, and provided with aseptic food and water. The animal experiments were approved by the Ethics Committee of Xinxiang Medical University.

The H22 tumor-bearing mice were established by subcutaneously injecting 2 × 10^6^ H22 cells into the right hind leg of C57BL/6 mice. After 7 days, the mice were randomly divided into five groups (5 mice/group), including a PBS group, a Scramble group, a radiation therapy group, a siRNA-PD-L1 group, and a radiation therapy combined with siRNA-PD-L1 group. The treatments commenced when the tumors had grown to approximately 100 mm^3^. Radiotherapy was performed in vitro using a 6 MeV electron beam linear accelerator (Elekta, Stockholm, Sweden) to deliver a dose of 4 Gy on the 10th day, while the recombinant attenuated *Salmonella* containing Scramble or siRNA-PD-L1 (4 × 10^5^ colony-forming units (CFU) in 100 μL PBS/mouse) were intratumorally injected twice on the 12th and 18th days.

### Colony formation assay

The tumor, heart, liver, spleen, lung, and kidney tissues were aseptically collected and ground (100 mg) and resuspended in 3 ml of PBS. Next, 300 μL of supernatant was transferred to a fresh tube and mixed thoroughly with 700 μL of PBS. The resulting mixture was inoculated onto LB solid medium with 100 μg/mL ampicillin and incubated overnight at 37 °C. The bacterial colonies formed on the following day were counted and quantitatively analyzed.

### Western blot

Total protein was extracted using RIPA lysate (Beyotime Institute of Biotechnology, Shanghai, China). Proteins were then separated by electrophoresis using 10% SDS/PAGE gels and transferred to a PVDF membrane (EMD Millipore, Billerica, MA, USA). The membrane was blocked with 5% defatted milk powder/PBS containing 0.05% Tween (PBST) for 2 h. After blocking, the membranes were incubated with specific primary antibodies (PD-L1, 1:1000, Bioworld, BS6850; p-Stat3, 1:1000, CST, 9145S; cyclin D1, 1:2000, SANTA, sc-56302; Bcl-2, 1:2000, Abways, CY5032; Cytochrome C, 1:1000, Biopple, CY5734; C-caspase3, 1:1000, CST, 9661S; C-caspase9, 1:2000, Abways, CY5682; Tubulin, 1:1000, Sigma, T8203) at 4 °C overnight. Secondary antibody (ZSGB-BIO, 1∶5000, ZB-2301 and ZB-2305) was incubated after finishing the incubation of the primary antibody. The immunoreactive bands were detected by a chemiluminescence page kit (Beyotime Institute of Biotechnology) and visualized by a chemiluminescence imager (Fusion FX spectra, Vilber). Lastly, the blots were analyzed using QuantaSoft Software (Version 4.62; Bio-Rad Laboratories, Inc., Hercules, CA, USA).

### Immunohistochemical (IHC)

The tumor tissues, fixed in 4% methanol, underwent dehydration and paraffin embedding and were cut into sections with a thickness of 4 μm. Subsequently, these sections were exposed to primary antibodies, including Ki67 (1:200, Bioworld, BS6667), PCNA (1:200, SANTA, sc-25280), and C-caspase3 (1:200, CST, 9661S), overnight at 4 °C. The following day, the sections were washed and then subjected to a 30-min incubation with biotinylated secondary antibody (catalog numbers PV-9001 and PV-9002 from ZSGB-BIO). After being washed with PBS, horseradish peroxidase was added to the sections for labeling, followed by color development through diaminobenzidine (DAB) for 5 min. Finally, the sections were monitored under a digital slide scanner (3DHISTECH, Pannoramic MIDI, China).

### Immunofluorescence (IF)

The tumor tissues, fixed with 4% methanol, underwent dehydration and paraffin embedding and were sliced into sections 4-μm thick. These sections were incubated overnight at 4 °C with primary antibodies (CD3, 1:100, OmnimAbs, OM244830; CD4, 1:200, CST, 25229S; CD8, 1:800, CST, 98941S; Granzyme B, 1:200, Bioworld, BS1152; CD86, 1:400, Novus, NBP2-25208; CD11b, 1:200, Abways, CY5019; PD-L1, 1:200, Bioworld, BS6850). Following the overnight incubation, the sections were washed and incubated with fluorophore-conjugated secondary antibodies (cat. AB0132, AB0121, Abways) diluted in blocking solution. After washing away the secondary antibodies, the slides were treated with DAPI (Beyotime) for 5 min and covered with an anti-fluorescence quenching reagent (Beyotime). The resulting images were then captured using confocal microscopy (AR1+, Nikon).

### TUNEL assay

The TUNEL assay was performed by employing the TUNEL detection kit (Beyotime Institute of Biotechnology, Shanghai, China) as per the manufacturer’s instructions. Following dewaxing and rehydration, tissue sections were permeabilized with proteinase K (DNase-free) and incubated at 37 °C for 20 min. Subsequently, the sections were washed with PBS and TUNEL detection solution was added. The nuclei were counterstained with DAPI for 5 min, and images were captured utilizing confocal microscopy (AR1+, Nikon).

### Flow cytometry

Spleen cell suspensions were prepared, and erythrocytes were eliminated utilizing a red blood cell lysis buffer (manufactured by the esteemed Beyotime Institute of Biotechnology, located in Shanghai, China). The cell concentration was precisely adjusted to 1 × 10^7^ cells/mL. Afterward, a 100 µL cell suspension was diligently incubated with the respective fluorochrome-labeled CD3 (cat. 100204), CD4 (cat. 100408), CD8 (cat. 100724), and NK 1.1 (cat. 108706) antibodies (all obtained from the prestigious BioLegend, Inc., based in Santiago, USA) in a dimly lit environment at a temperature of 4 °C for 30 min. Following this, the cells were thoroughly washed with PBS, and the fluorescence was detected by a top-of-the-line Cyto FLEX flow cytometer (manufactured by the renowned Beckman Coulter, situated in Miami, FL, USA).

### Statistical analysis

The statistical analysis of data was carried out using the SPSS 24.0 software. The measurement data, which adhered to the normal distribution, were presented as the mean ± standard deviation (mean ± SD). To examine the differences among the various groups, one-way ANOVA was performed. A statistically significant difference was considered at a level of *P* < 0.05.

### Supplementary information

Original blots for WB

## Data Availability

All data needed to evaluate the conclusions in the paper are present in this article or the supplementary materials. All materials may be made available to the scientific community upon request.
