# Supplementary figures and images for: Attenuated Salmonella carrying siRNA-PD-L1 and radiation combinatorial therapy induces tumor regression on HCC through T cell-mediated immuno-enhancement
Source: Cell Death Discov. 2023 Aug 28;9:318. doi: 10.1038/s41420-023-01603-x (PMC10462685; doi:10.1038/s41420-023-01603-x)

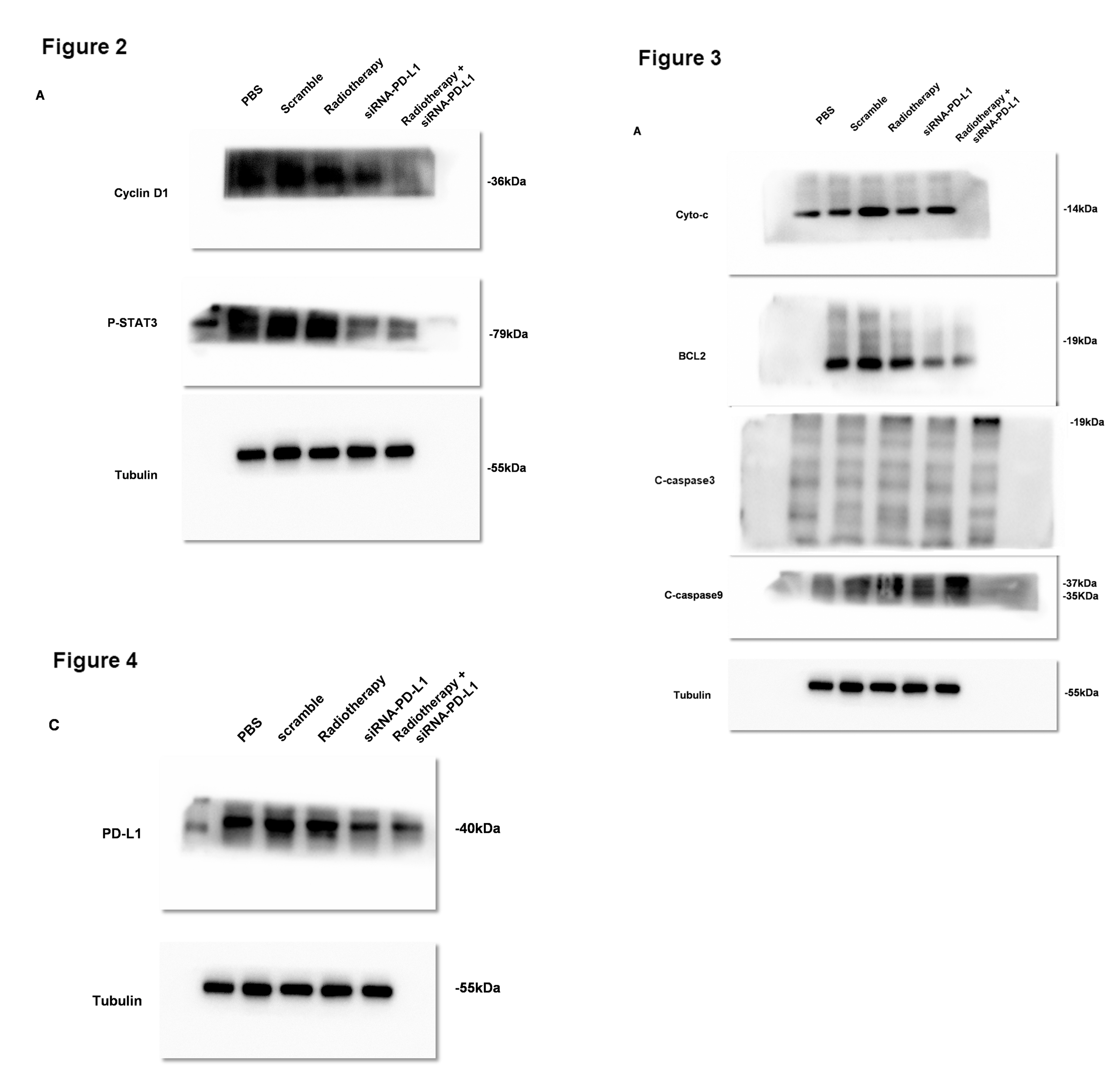

Supplement: Supplementary file 1 — Original blots for WB [file 41420_2023_1603_MOESM1_ESM.tif]
